# Synovial fluid specific gravity as an inexpensive point-of-care test for diagnosing hip and knee periprosthetic joint infection

**DOI:** 10.5194/jbji-11-15-2026

**Published:** 2026-01-12

**Authors:** Sujeesh Sebastian, Hibah A. Abusulaiman, Veronika Achatz, Matteo Spadini, Jennyfer A. Mitterer, Sebastian Simon, Jochen G. Hofstaetter

**Affiliations:** 1 Michael Ogon Laboratory for Orthopaedic Research, Orthopaedic Hospital Vienna-Speising, Speisinger Straße 109, 1130 Vienna, Austria; 2 Department of Orthopaedics and Trauma, Medical University of Graz, Auenbruggerplatz 5, Graz, Austria; 3 2nd Department, Orthopaedic Hospital Vienna-Speising, Speisinger Straße 109, 1130 Vienna, Austria

## Abstract

Synovial fluid specific gravity (SG) was evaluated as a rapid, inexpensive test for periprosthetic joint infection (PJI) diagnosis in revision arthroplasties. High diagnostic accuracy (area under the curve 0.89; threshold 1.007) with high specificity (100 %) and moderate sensitivity (65 %) was found, supporting its use as an adjunctive point-of-care (POC) tool for PJI.

## 1 Introduction

Synovial fluid analysis plays a crucial role in the diagnosis of periprosthetic joint infection (PJI) (Shohat et al., 2019; McNally et al., 2021). Although current established synovial fluid inflammatory markers for PJI, such as synovial white blood cell (WBC) count or polymorphonuclear leukocyte percentage (PMN %), provide valuable information, they are often either not readily available intraoperatively or in outpatient clinics. Additionally, novel synovial fluid biomarkers such as alpha-defensin are expensive, unsuitable for resource-limited hospitals, and offer limited diagnostic value over standard tests, making routine use unnecessary (Li et al., 2023; Amanatullah et al., 2020). Therefore, there is a need to explore more rapid and inexpensive point-of-care (POC) tests for PJI.

In septic joints and PJIs, the composition of synovial fluid changes substantially compared with non-infectious effusions (de Paula Mozella et al., 2024; Sowislok et al., 2024; Sebastian et al., 2025). The specific gravity (SG) of synovial fluid reflects the combined concentration of cellular elements (predominantly neutrophils and other leukocytes), plasma-derived proteins (albumin, immunoglobulins, fibrinogen), locally produced acute-phase and defence proteins, lipoproteins, and bacterial products within the joint space (MacWilliams and Friedrichs, 2003; Faryna and Goldenberg, 1990; Yehia and Duncan, 1975). Infected synovial fluid which is characterized by high leukocyte burden and protein enrichment, as well as an altered metabolomic profile with increased unsaturated lipids and N-acetylated glycoproteins, contributes additional mass per unit volume and thereby increases SG relative to aseptic joints (de Paula Mozella et al., 2024; Akhbari et al., 2021; Sendi et al., 2018; Faryna and Goldenberg, 1990). Nevertheless, no previous studies have assessed this relationship or its potential for diagnosing PJI. In addition, SG levels in synovial fluid can easily be determined using a strip POC method that is inexpensive and readily accessible.

In this study, we aimed to evaluate whether synovial fluid SG levels can be used as an inexpensive POC test for diagnosing hip and knee PJI. In addition, we assessed the optimal threshold value, PJI diagnostic accuracy, and correlation with other biomarkers. We hypothesized that the level of synovial fluid SG would be higher in cases of PJI than in aseptic revisions and that it would be clinically useful in diagnosing hip and knee PJI.

## 2 Materials and methods

### 2.1 Study design and patient selection

A retrospective analysis of our prospectively maintained institutional arthroplasty registry and musculoskeletal biobank was done. All patients who underwent revision total hip and knee arthroplasty (rTHA and rTKA) between 2019 and 2024 were screened. Following European Bone and Joint Infection Society (EBJIS) PJI criteria, the revision procedures were classified as “infection confirmed” or “unlikely” (McNally et al., 2021). Clinical and baseline demographic data were collected for all patients, including age, sex, body mass index, arthroplasty localization, preoperative laboratory values (serum C-reactive protein, synovial fluid WBC, and PMN %), number of prior revision surgeries, microbiology, and pathology reports. Patients who underwent reimplantation and spacer exchange were excluded from the study.

### 2.2 Synovial fluid SG measurement

In accordance with institutional protocol, arthrocentesis was conducted under sterile conditions for all revision arthroplasties. In addition to standard cell count and microbiological analysis, surplus synovial fluid samples were preserved in our institutional musculoskeletal biobank at - 80 °C for subsequent analysis. To determine the SG, selected frozen synovial fluid samples were thawed at room temperature and processed using the Combur^10^ test UX strip method (Roche Diagnostics GmbH, Mannheim, Germany) following the manufacturer's instructions. Briefly, a synovial fluid volume of 10 µ L per test was placed on the strip's SG detection pad, and after 1 min, the pad's colour was compared with the colour scale value (range: 1.000–1.030) on the test strip container.

### 2.3 Data analyses

For categorical variables, frequencies were computed, while for continuous variables, means or medians with confidence intervals or percentiles were calculated as appropriate. The Pearson's chi-squared test and Mann–Whitney U test were employed to compare clinical characteristics and biomarker findings between infected and non-infected cases for categorical and continuous variables, respectively. The diagnostic accuracy of SG was evaluated using the area under the receiver operating characteristic (ROC) curve, sensitivity, specificity, positive predictive values, and negative predictive values. Optimal cutoff values were determined by Youden's index. To calculate diagnostic accuracy, we initially conducted an optimal performance analysis by excluding tests that were unreadable due to blood-contaminated synovial fluid. Subsequently, SG's performance in the intention-to-diagnose scenario was assessed by considering the unreadable tests as negative results, using a cutoff lower than that derived from the optimal performance analysis. Spearman correlation analysis was used to determine the association between SG and other inflammatory biomarkers. Statistical significance was set at P<0.05 . All analyses were conducted using Statistical Package for Social Sciences (SPSS) 25 (IBM Corporation, released 2018, IBM SPSS Statistics for Windows, Version 25.0., Armonk, NY).

## 3 Results

### 3.1 Study population and revision arthroplasty characteristics

A total of 228 patients (141 women, 87 men) who underwent 250 revision arthroplasties (120 rTHA, 130 rTKA) were included in the final analysis (Table 1). Of these 250 revision arthroplasties, 120 (50 rTHA and 70 rTKA) were classified as infection confirmed, and 130 (57 rTHA and 73 rTKA) were infection unlikely according to EBJIS criteria. The most frequently isolated pathogen in septic revisions was *Staphylococcus epidermidis* (20 %).

### 3.2 Diagnostic accuracy of synovial fluid specific gravity

Of the 250 samples analysed, SG levels were not detectable in 36 patients (infected = 15, infection unlikely = 21) who had blood mixed with synovial fluid. In the remaining patients, the distribution of synovial SG and other standard biomarkers differed significantly between septic and aseptic revision arthroplasties ( P<0.001 ) (Table 1).

In the analysis of optimal performance, SG demonstrated an excellent AUC (0.89), and the performance was slightly better in knee cases (AUC 0.91) than in hip cases (AUC 0.87) (Table 2). When failed tests were included as negative in an intention-to-diagnose analysis, the AUC decreased from 0.89 (95 % CI 0.85–0.94) to 0.83 (95 % CI 0.78–0.88), and the decrease in AUC did not reach statistical significance ( P=0.09 ).

### 3.3 Relation between specific gravity and other biomarkers

Spearman correlation analysis identified significant positive associations between SG and other inflammatory biomarkers. SG demonstrated moderate to strong correlations with serum CRP ( r=0.646 , P<0.001 ), synovial WBC ( r=0.711 , P<0.001 ), and PMN % ( r=0.685 , P<0.001 ).

**Table 1:** Demographic and clinical data.

	rTHA				rTKA			
	Total	Infection confirmed	Infection unlikely	P value	Total	Infection confirmed	Infection unlikely	P value
Total procedures, n (%)	107	50 (47)	57 (53)	0.628	143	70 (49)	73 (51)	0.720
Sex, n (%)				0.068				0.062
Female	62 (61.4)	25 (52)	37 (70)		79 (62.2)	31 (53.4)	48 (69.6)	
Male	39 (38.6)	23 (48)	16 (30)		48 (37.8)	27 (46.6)	21 (30.4)	
Total patients	101	48	53		127	58	69	
Age in years (mean ± SD)	70 ± 13.3	70.3 ± 12.6	69.9 ± 14.0	0.992	68.9 ± 11	70.7 ± 8.9	67.4 ± 12.4	0.274
BMI (mean ± SD)	28.6 ± 5.9	30.3 ± 6.9	26.9 ± 4.4	0.006	30.6 ± 7.4	30.5 ± 7.4	30.6 ± 7.4	0.940
Serum and synovial inflammatory markers (median and IQR)								
seCRP (mg L^−1^)	6.6 (2.0–39.7)	47.1 (8.8–130.3)	2.6 (1.3–6.3)	< 0.001^*^	9.9 (2.5–79.7)	79.4 (31.7–175.6)	2.5 (1–4)	< 0.001^*^
syWBC count (cells µ L^−1^)	1780 (645–25 370)	23 250 (6010–73 350)	645 (340–1048)	< 0.001^*^	1440 (340–37 950)	38 485 (14 225–67 755)	360 (200–710)	< 0.001^*^
syPMN ( %)	54.4 (29.4–88.4)	87.7 (61.7–94.8)	29.4 (14–45)	< 0.001^*^	50.9 (22.6–88.6)	88.7 (83.4–94.1)	22.7 (15.6–30.8)	< 0.001^*^
SG	1.005 (1.000–1.010)	1.010 (1.005–1.015)	1.000 (1.000–1.005)	< 0.001^*^	1.005 (1.000–1.010)	1.010 (1.005–1.013)	1.000 (1.000–1.005)	< 0.001^*^
On antibiotic therapy at the time of aspiration, n (%)	9 (8.4)	9 (18)	0	< 0.001^*^	19 (13.3)	19 (27)	0	< 0.001^*^
Type of revision				0.426				0.929
1st revision	60 (56)	26 (52)	34 (60)		70 (49)	34 (48.6)	36 (49.3)	
Re-revision	47 (44)	24 (48)	27 (40)		73 (51)	36 (51.4)	37 (50.7)	
Time to previous surgery				< 0.001^*^				< 0.001^*^
< 90 d (acute procedure)	28 (26)	24 (48)	4 (7)		15 (11)	14 (20)	1 (1.4)	
> 90 d (chronic procedure)	79 (74)	26 (52)	53 (93)		128 (90)	56 (80)	72 (98.6)	
ASA score, n (%)				0.109				0.012
ASA I	14 (13.1)	5 (10)	9 (15.8)		7 (4.9)	2 (2.9)	5 (6.8)	
ASA II	72 (67.3)	31 (62)	41 (71.9)		102 (71.3)	44 (62.9)	58 (79.5)	
ASA III	21 (19.6)	14 (28)	7 (12.3)		33 (23.1)	24 (34.3)	9 (12.3)	
ASA IV	0	0	0		1 (0.7)	0	1 (1.4)	
Charlson comorbidity index, median (range)	3 (0–10)	3.5 (0–10)	3 (0–8)	0.569	4 (0–10)	4 (1–10)	3 (0–9)	0.128

rTHA, revision total hip arthroplasty; rTKA, revision total knee arthroplasty; SD, standard deviation; BMI, body mass index, IQR, interquartile range; seCRP, serum C-reactive protein; syWBC, synovial white blood cell; syPMN, synovial polymorphonuclear leukocytes; SG, specific gravity; ASA, American Society of Anesthesiologists; ^*^ significant difference.

**Table 2:** Diagnostic accuracy of synovial fluid specific gravity assessed through receiver operating curve analysis, conducted via (A) optimal performance analysis, which excludes non-readable tests due to blood-contaminated synovial fluid, and (B) intention-to-diagnose analysis, where non-readable tests were considered to be negative results.

Specific gravity	AUC (95 % CI)	Threshold	Sensitivity	Specificity	ppv	npv
(A) Optimal performance analysis						
Hip and knee	0.89 (0.85–0.94)	1.007	0.65	1.00	1.00	0.75
– Hip	0.87 (0.80–0.94)	1.007	0.58	1.00	1.00	0.69
– Knee	0.91 (0.85–0.96)	1.007	0.70	1.00	1.00	0.79
(B) Intention-to-diagnose analysis						
Hip and knee	0.83 (0.78–0.88)	1.007	0.57	1.00	1.00	0.71
– Hip	0.87 (0.80–0.94)	1.007	0.56	1.00	1.00	0.72
– Knee	0.81 (0.73–0.88)	1.007	0.57	1.00	1.00	0.71

AUC, area under the curve; CI, confidence interval; ppv, positive predictive value; npv, negative predictive value

## 4 Discussion

Our results suggest that synovial fluid SG levels can be used as an inexpensive POC diagnostic test for PJI in the hip and knee revision arthroplasties. Although SG demonstrated moderate sensitivity, it exhibited excellent specificity and overall good diagnostic accuracy for PJI. The strong correlations observed with established inflammatory biomarkers further support SG as an adjunctive marker for PJI detection.

In the literature, only a limited number of studies have investigated the utility of alternative physical properties of synovial fluid, like viscosity for the diagnosis of PJI, with none focusing on synovial SG (Roškar et al., 2025; Fu et al., 2019). The reported AUC of synovial viscosity was 0.83, and, in our study, synovial SG demonstrated an overall AUC of 0.89, indicating its excellent diagnostic performance (Roškar et al., 2025). The specificity of 100 % suggests that a positive SG result is highly reliable for confirming PJI. However, the low sensitivity of 65 % indicates that SG is more suitable as a rule-in rather than a rule-out test. Consequently, SG can complement but not replace recommended PJI markers. While the reduction in AUC and sensitivity in the intention-to-diagnose analysis was not statistically significant, this finding suggests the limited utility of SG strips in synovial fluid samples contaminated with blood. It also introduces uncertainty regarding their applicability across all revision surgeries, thereby necessitating further methodological optimization. Nevertheless, its low cost (less than EUR 1 per test), ability to deliver immediate results (1 min), and minimal sample volume requirement (10 µ L per test) could make it ideal for intraoperative use or in outpatient clinics, as well as in resource-limited countries with inadequate laboratory infrastructure.

While it is evident that infections increase synovial SG by activating inflammatory cells and other proteins, we observed a strong correlation between SG and other major biomarkers (Akhbari et al., 2021; Faryna and Goldenberg, 1990). Our findings therefore indicate that both local cellular inflammations, such as WBC or PMN %, and systemic inflammatory burden, as indicated by CRP, are crucial determinants of synovial SG, thus capturing a broad spectrum of infection-related changes. In the diagnostic context, this may be valuable, unlike costly biomarkers such as alpha-defensin, which measures only a single neutrophil-derived peptide (Amanatullah et al., 2020). However, given that its measurement can be influenced by multiple inflammatory signals, further research is necessary to elucidate the additional synovial fluid components contributing to its elevation during PJI.

The present study acknowledges a few limitations. The utilization of SG test strips, originally designed for urine diagnostics, in the context of PJI with minimal modification or validation for synovial fluid may impact the generalizability of our results. Nonetheless, this study is one of the first to investigate the SG as a potential marker for PJI, and our findings offer further insights into the use of synovial physical properties as simple and easy-to-use diagnostic markers. SG measurement was performed on stored frozen synovial samples rather than fresh aspirations. This underscores the necessity for prospective evaluation in large, multi-centre cohorts to validate optimal threshold values and assess real-time performance. Finally, although red blood cell (RBC) contamination from unstable joints represents a significant pre-analytical confounder for strip-based SG measurement, this study did not conduct a systematic RBC quantification and subgroup analysis of RBC versus WBC contributions to synovial SG changes, warranting separate evaluation in future validation studies.

## 5 Conclusions

In conclusion, synovial fluid SG is a promising and inexpensive POC biomarker for the diagnosis of hip and knee PJI. Its strong correlations with established markers and high diagnostic accuracy highlight its robustness as a complementary diagnostic test. However, due to its moderate sensitivity and limited utility in blood-contaminated synovial fluid samples, further prospective studies are required to validate our findings and to assess its clinical applicability in the early detection of PJI. 

## Data Availability

Data are available upon request.
